# Examining Disparities in Access to Kidney Transplant Listing Before and During the COVID-19 Pandemic

**DOI:** 10.7759/cureus.102327

**Published:** 2026-01-26

**Authors:** Joseph M Cotton, Silke Niederhaus, Keisha Perry, Nadiesda Costa, Jonathan Bromberg, Raphael Meier

**Affiliations:** 1 Surgery, University of Maryland School of Medicine, Baltimore, USA; 2 Transplant Nephrology, MedStar Georgetown University Hospital, Washington, DC, USA

**Keywords:** covid-19, employment, ethnicity, kidney, pandemic, retrospective, socioeconomic factors, telemedicine

## Abstract

Introduction: Despite high dialysis prevalence, access to kidney transplant waitlisting remains limited, with persistent socioeconomic and racial disparities. Prior studies have not captured patients evaluated but not listed for transplantation. During the COVID-19 pandemic, telemedicine-based evaluations may have further widened these gaps. We examined transplant evaluation and waitlisting before and during the pandemic at a large urban transplant center.

Purpose: We hypothesized that the COVID-19 pandemic would adversely affect access to listing for kidney transplant (KT) for transplant candidates from minority ethnic groups. We evaluated differences in listing for kidney transplant between pre- and peri-pandemic eras, in-person (IPE) and telemedicine (TME) evaluations, and associations with race/ethnicity and other socioeconomic factors.

Methods: A retrospective chart review of 1061 KT evaluations included pre-pandemic (March 13, 2019, to March 13, 2020) and peri-pandemic (March 14, 2020, to March 14, 2021) eras. Categorical data are presented as proportions and frequencies, and continuous data as means ± standard deviation or medians ± interquartile range. Independent group t-tests and Fisher’s exact tests were used for bivariate comparisons.

Results: A total of 629 (59%) candidate evaluations were pre-pandemic, and 430 (41%) were peri-pandemic. Of these, 734 (72%) were IPE and 288 (28%) were TME. Overall, 553 (54%) candidates were denied listing for medical (310) and social (184) reasons. Peri-pandemic evaluations (p = 0.002), employment (p < 0.001), TME (p < 0.001), and mental health (p = 0.009) were associated with listing. Positive assessments of social support and overall social work assessment were associated with listing (p = 0.002; p < 0.001). Better social support was associated with listing pre-pandemic (p = 0.001) but not peri-pandemic (p = 0.769). Race/ethnicity (p = 0.951), employment (p = 0.202), and mental health (p = 0.742) were similar pre- and peri-pandemic. Race/ethnicity was not associated with listing (p = 0.809).

Conclusion: Overall, more candidates were listed for KT during the pandemic than before, despite fewer evaluations. This increase was associated with TME but not with race/ethnicity. Employment and mental health were similar in both eras despite pandemic stressors. Denial for social reasons mostly affected minority candidates, which warrants further study.

## Introduction

Access to kidney transplantation is a major healthcare concern across the United States. Currently, there are 468,000 people on dialysis in the United States [1]. However, only 109,000 people are listed for kidney transplants as of September 2020 [2]. This study aims to examine this discrepancy more closely. The University of Maryland Medical Center is an optimal setting for this study because of its location and volume of transplants performed each year. Because this center is located in Baltimore, it receives a diverse group of patients, many of whom have been affected by pre-existing Redlining laws [3] and neighborhood segregation.

Many studies have sought to explain the discrepancy between individuals on dialysis and individuals on the transplant waiting list. Peng [4] and colleagues examined the association between neighborhood characteristics and racial disparities in reaching the kidney transplant waiting list. As explained by the authors, this study is limited by its lack of information regarding patient socioeconomic status. In addition, Peng’s study did not examine patients who were evaluated for kidney transplants and were never listed. Instead, the authors examined all patients on dialysis who had not been waitlisted. Our study aims to examine the population of end-stage renal disease patients who were evaluated but not listed for kidney transplantation, in comparison to those who were. These data are not reported centrally, and thus no nationwide data exist regarding transplant evaluations, only waitlist additions and removals.

Hospitals across the country have seen a tremendous increase in their use of telemedicine as a result of the pandemic. Wangberg [5] and colleagues illustrated how internet access varies across different socioeconomic statuses. They found that internet access has a positive association with both subjective health and socioeconomic status. This association led us to hypothesize that access to telemedicine in the COVID-19 pandemic may disproportionately affect different ethnic subgroups with regard to access to the kidney transplant waitlist.

Prolonged dialysis time and decreases in access to transplant waitlists are both associated with poor prognosis for individuals with chronic kidney disease. The COVID-19 pandemic may have further complicated access to kidney transplantation for socially disadvantaged groups due to the transition from in-person evaluation (IPE) to telemedicine evaluations (TME), which may not have been accessible equally to all transplant candidates. We thus decided to evaluate differences in evaluation and listing of candidates for kidney transplant before and during the COVID-19 pandemic. This article was previously presented as a meeting abstract at the 2022 Annual American Transplant Congress on June 7, 2022.

## Materials and methods

In our retrospective cohort study, we analyzed individuals aged 18 and above who underwent evaluations for kidney and kidney-pancreas transplants at the University of Maryland Medical Center. Our study spanned two crucial periods: Group 1, which encompassed assessments conducted from March 13, 2019, to March 13, 2020, representing the pre-pandemic era, and Group 2, comprising evaluations carried out from March 14, 2020, to March 14, 2021, representing the peri-pandemic phase. Notably, we excluded re-evaluations from our analysis, focusing solely on outcomes derived from the initial assessments aimed at determining listing candidacy.

To ascertain the rationale behind candidacy denials, we examined notes authored by social workers affiliated with the University of Maryland Medical Center. Their documentation provided insights into the factors influencing listing decisions. This research endeavor received full approval from the University of Maryland School of Medicine Institutional Review Board, ensuring adherence to ethical standards and protocols. We aimed to shed light on the impact of the COVID-19 pandemic on transplant candidacy assessments, thereby contributing to the broader understanding of healthcare dynamics during this unprecedented period.

Data collection

Data for this retrospective cohort study were obtained through detailed chart review of adult patients (≥18 years) who underwent initial evaluation for kidney or kidney-pancreas transplantation at the University of Maryland Medical Center. Demographic variables collected included age, sex, race/ethnicity, employment status, insurance type, and distance from the transplant center. Clinical data included cause of kidney disease, transplant type, mental health history, and active substance use. Evaluation-related variables included evaluation era (pre-pandemic vs peri-pandemic), mode of evaluation (in-person vs telemedicine), and transplant listing outcome. Social determinants of health were abstracted from transplant social work assessments documented in the electronic medical record and included level of social support, overall social work assessment, and specific reasons for denial when applicable. Reasons for denial were categorized as medical or social based on social worker documentation. All variables were abstracted using a standardized data collection approach to ensure consistency across records, and only data from initial transplant evaluations were included; re-evaluations were excluded.

Statistical analyses

Characteristics of the overall transplant candidate cohort were reported as mean and standard deviation, median and interquartile range, or counts and percentages, as appropriate. The sample size consisted of 1061 transplant candidates. To analyze the relationship between independent variables and the primary outcome (listing for kidney or kidney-pancreas transplant), multivariate analyses were performed using chi-square or Fisher’s exact test for categorical variables and Wilcoxon rank sum testing (or Kruskal-Wallis testing) for continuous variables, as appropriate. All statistical tests were two-tailed and utilized a 5% significance level. Statistical analysis was performed using STATA software version 9.4 (StataCorp. 2009. Stata Statistical Software: Release 11. College Station, Texas).

## Results

A total of 1061 records were reviewed. Data were available in over 90% of patients for all variables studied. Overall, our study population was quite diverse, with 60% of transplant candidates identifying as African American or Black, 33% as Caucasian or White, 4% as Hispanic, and 3% as Asian (Table 1).

**Table 1:** Evaluations by era

Variables	Overall, n (%)	Pre-pandemic, n (%)	Peri-pandemic, n (%)	P-value
Race and ethnicity (N = 1015)				
African American/Black	608 (60%)	359 (59%)	249 (61%)	0.951
Caucasian/White	335 (33%)	199 (33%)	136 (33%)	
Hispanic	40 (4%)	25 (4%)	15 (4%)	
Asian	29 (3%)	19 (3%)	10 (2%)	
Other	3 (0%)	2 (<1%)	1 (<1%)	
Cause of kidney disease (n=960)				0.007
Hypertension and diabetes	288 (30%)	197 (34%)	91 (24%)	
Hypertension	286 (30%)	166 (29%)	120 (31%)	
Diabetes	156 (16%)	91 (16%)	65 (17%)	
Polycystic kidney disease	40 (4%)	26 (5%)	14 (4%)	
Drug-induced	37 (4%)	20 (4%)	17 (4%)	
Lupus	24 (3%)	14 (2%)	10 (3%)	
Other	129 (13%)	62 (11%)	67 (17%)	
Employment status (N = 979)				0.202
Employed	278 (28%)	162 (27%)	116 (30%)	
On disability	368 (38%)	220 (37%)	148 (38%)	
Unemployed	66 (7%)	35 (6%)	31 (8%)	
Retired	267 (27%)	174 (29%)	98 (24%)	
Type of evaluation (N = 1022)				<0.0001
In-person evaluation	734 (72%)	604 (99.8%)	130 (31.1%)	
Telemedicine evaluation	288 (28%)	1 (0.2%)	287 (69.9%)	
Distance to transplant center (N = 971)				0.343
0-30 miles (public transportation)	529 (54%)	323 (55%)	206 (51%)	
31-100 miles (easy drive)	279 (30%)	171 (29%)	126 (31%)	
>100 miles (long-distance)	163 (16%)	90 (15%)	73 (18%)	
Type of insurance (N = 1000)				0.143
Commercial	893 (89%)	527 (88%)	366 (91%)	
Private	107 (11%)	71 (12%)	36 (9%)	
Evaluation outcome (N = 1022)				0.002
Accepted for listing	469 (46%)	253 (42%)	216 (52%)	
Denied for listing	553 (54%)	352 (58%)	201 (48%)	
Level of social support (N = 963)				0.564
Excellent	360 (37%)	214 (36%)	146 (39%)	
Good	426 (44%)	270 (41%)	156 (42%)	
Moderate	137 (14%)	80 (14%)	57 (15%)	
Limited	32 (3%)	22 (4%)	10 (3%)	
Poor	8 (<1%)	4 (<1%)	4 (1%)	
Social work assessment (N = 969)				0.005
Excellent candidate	304 (31%)	178 (30%)	126 (33%)	
Good candidate	517 (53%)	302 (51%)	215 (57%)	
Minimally acceptable candidate	125 (13%)	90 (15%)	35 (9%)	
Poor candidate	23 (2%)	19 (3%)	4 (1%)	
Mental health status (N = 964)				0.742
No psychopathology	643 (67%)	385 (65%)	258 (69%)	
Mild psychopathology	231 (24%)	146 (25%)	85 (23%)	
Moderate psychopathology	70 (7%)	44 (7%)	26 (7%)	
Severe psychopathology	18 (2%)	13 (2%)	5 (1%)	
Extreme psychopathology	1 (<1%)	1 (<1%)	2 (<1%)	
Active substance use (N = 981)				0.100
None	754 (77%)	462 (79%)	292 (74%)	
Alcohol	144 (15%)	72 (12%)	72 (18%)	
Marijuana	34 (3%)	23 (4%)	11 (3%)	
Tobacco	42 (4%)	26 (4%)	16 (4%)	
Cocaine	7 (<1%)	5 (<1%)	2 (<1%)	
Transplant number (N = 1019)				0.472
First	921 (90%)	542 (90%)	379 (91%)	
Second	93 (9%)	59 (10%)	34 (8%)	
Third	5 (<1%)	2 (<1%)	3 (<1%)	

Comparison of pre- and peri-pandemic eras

When comparing the pre- and peri-pandemic eras, we noted differences in the cause of kidney disease and social work assessment of candidates (Table 1). Overall, fewer evaluations took place in the peri-pandemic era (n = 417) compared to the pre-pandemic era (n = 605); however, acceptance for listing was significantly higher in the peri-pandemic era (p = 0.002, Table 1). In the peri-pandemic era, more evaluations took place via TME (p < 0.0001) compared to the pre-pandemic era (Table 1). In fact, only a single evaluation was performed by telemedicine prior to the pandemic.

No differences were noted between the pre- and peri-pandemic eras in terms of race and ethnicity of candidates evaluated, employment status, distance to the transplant center, type of insurance, level of social support, mental health status, active substance use, or transplant number (Table 1).

Association between telemedicine and acceptance for listing

During the peri-pandemic era, 287 candidates were seen by telemedicine, of whom 158 (55%) were accepted for listing. This compares with a 45% acceptance rate for patients seen in person in the peri-pandemic era, and an overall acceptance rate for all candidates of 46% (Tables 1, 2).

**Table 2:** Acceptance for listing overall

Variables	Overall, n (%)	Accepted, n (%)	Denied, n (%)	P-value
Race and ethnicity (N = 1013)				0.809
African American/Black	607 (60%)	284 (61%)	323 (59%)	
Caucasian/White	334 (33%)	147 (32%)	187 (34%)	
Hispanic	40 (4%)	18 (4%)	22 (4%)	
Asian	29 (3%)	15 (3%)	14 (2%)	
Other	3 (<1%)	2 (<1%)	1 (<1%)	
Cause of kidney disease (N = 957)				<0.0001
Hypertension and diabetes	288 (30%)	100 (23%)	188 (36%)	
Hypertension	285 (30%)	145 (33%)	140 (27%)	
Diabetes	156 (16%)	72 (16%)	84 (16%)	
Polycystic kidney disease	40 (4%)	27 (6%)	13 (3%)	
Drug-induced	37 (4%)	16 (4%)	21 (4%)	
Lupus	24 (3%)	15 (3%)	9 (2%)	
Other	127 (13%)	66 (15%)	61 (2%)	
Employment status (N = 979)				<0.0001
Employed	278 (28%)	178 (40%)	100 (20%)	
On disability	368 (38%)	162 (36%)	206 (39%)	
Unemployed	66 (7%)	33 (7%)	33 (6%)	
Retired	267 (27%)	77 (17%)	190 (36%)	
Type of evaluation (N = 1019)				<0.0001
In-person evaluation	732 (72%)	310 (66%)	422 (77%)	
Telemedicine evaluation	287 (28%)	158 (34%)	129 (23%)	
Distance to transplant center (N = 986)				0.693
0-30 miles (public transportation)	528 (54%)	236 (52%)	292 (55%)	
31-100 miles (easy drive)	296 (30%)	141 (31%)	155 (29%)	
>100 miles (long-distance)	162 (16%)	76 (17%)	86 (16%)	
Type of insurance (N = 1000)				0.018
Commercial	893 (89%)	492 (91%)	401 (87%)	
Private	107 (11%)	46 (9%)	61 (13%)	
Level of social support (N = 963)				0.002
Excellent	360 (37%)	174 (39%)	186 (36%)	
Good	426 (44%)	196 (44%)	230 (45%)	
Moderate	137 (14%)	58 (13%)	79 (15%)	
Limited	32 (3%)	5 (1%)	27 (5%)	
Poor	8 (<1%)	3 (<1%)	5 (1%)	
Social work assessment (N = 969)				<0.0001
Excellent candidate	304 (31%)	165 (36%)	139 (27%)	
Good candidate	517 (53%)	258 (57%)	259 (50%)	
Minimally acceptable candidate	125 (13%)	30 (7%)	95 (18%)	
Poor candidate	23 (2%)	1 (2%)	22 (4%)	
Mental health status/psychopathology (N = 964)				0.009
None	643 (67%)	315 (70%)	328 (64%)	
Mild	231 (24%)	107 (24%)	124 (24%)	
Moderate	70 (7%)	21 (5%)	49 (10%)	
Severe	18 (2%)	5 (1%)	13 (3%)	
Extreme	2 (<1%)	0 (0%)	2 (<1%)	
Active substance use (N = 978)				0.472
None	751 (77%)	344 (76%)	407 (77%)	
Alcohol	144 (15%)	74 (16%)	70 (13%)	
Marijuana	34 (3%)	13 (3%)	21 (4%)	
Tobacco	42 (4%)	18 (4%)	24 (5%)	
Cocaine	7 (<1%)	2 (<1%)	5 (<1%)	
Transplant number (N = 1016)				0.190
First	918 (90%)	415 (90%)	503 (91%)	
Second	93 (9%)	47 (10%)	46 (8%)	
Third	5 (<1%)	4 (<1%)	1 (<1%)	

Comparison between candidates accepted for listing and those denied listing for a kidney or kidney-pancreas transplant

Overall, cause of kidney disease, employment status, TME (vs IPE), type of insurance, a higher level of social support, better social work assessment, and better mental health status were associated with being listed for transplant (Table 2). Notably, there were no differences in listing due to race/ethnicity, distance to the transplant center, active substance use, or transplant number (Table 2).

Denials for listing

Lastly, we examined specifically those candidates who were denied access to the transplant list after their initial evaluation for kidney or kidney-pancreas transplant. Overall, there were 547 denials, including those that were deferred; 494 of these had data on the reason for denial. Of these, 310 (56%) were for medical reasons and 184 (33%) for social reasons. Furthermore, more African American candidates were denied for social reasons than medical reasons when compared to any other race.

## Discussion

Evaluations for kidney transplant at our center decreased by about one-third during the peri-pandemic era. The prevalence of kidney disease did not decrease during this time. In 2019, over 130,000 patients were diagnosed with end-stage renal disease (ESRD), which is an increase of 2.5% from the year prior [6]. Therefore, while ESRD continued to increase, fewer patients were being evaluated for transplant. Kidney transplants can increase life expectancy by at least 10 years [7]. We do not have data on patients with kidney disease who were not evaluated, but future studies should examine the impact of the decrease in evaluations on mortality from end-stage kidney disease. Simultaneous to a decrease in evaluations, there was an increase in acceptance for listing in the peri-pandemic era, with no change in racial or ethnic composition, and this increase was associated with TME. A recent study on the importance of technology access for successful telemedicine visits highlighted this disparity. Black, female, and Hispanic patients have decreased use of video for telehealth visits and increased use of telephone visits and are thus less likely to receive specialty care [8]. It is conceivable that some decrease in the number of evaluations in our study during the peri-pandemic era may have been due to a lack of patient access to telemedicine. Patient access to technology remains a significant barrier in healthcare [9].

Employment status and mental health status among kidney transplant candidates were similar in the pre- and peri-pandemic eras. This finding was surprising due to the additional stressors on employment and mental health attributed to the pandemic. According to data collected by the Congressional Research Service [10], the unemployment rate during the pandemic peaked at 14.8%, which is the highest rate since they began collecting data in 1948. This fact, coupled with higher rates of anxiety, depression, peri-traumatic stress disorder, and stress [11] observed during the pandemic, led us to assume that decreases in employment status and mental health status would be associated with the peri-pandemic era. One possible explanation for these contrary results from our study is the decrease in the total number of evaluations in the peri-pandemic era. Perhaps individuals of lower employment status and poorer mental health status did not present for transplant evaluation in the first place, thus skewing the data [12].

Additionally, race and ethnicity did not correlate with acceptance for listing when compared to denials for the kidney transplant waitlist, as we hypothesized. Previous studies have shown that African American patients are less likely than White patients to be rated as good candidates, placed on the transplant waiting list, and referred for evaluations [13]. Our findings are inconsistent with these previous studies. It is unclear whether this difference relates to our center’s location in particular; thus, our findings may not be broadly generalizable. Future studies are needed to allow comparisons between other transplant centers in locations different from ours. Candidates across each ethnic group were accepted vs denied at similar rates. However, employment status was a significant variable in predicting transplant acceptance. Candidates who were employed were accepted at a much higher rate than those in any other employment category. Similar to technology access, this is logical because employed candidates are more likely to be financially stable. This is consistent with findings from previous studies [14] that highlight the association between employment status and access to healthcare. Candidates who identified as retired were accepted at a lower rate than those in any other employment category. Retired candidates are more likely to be of older age, and older age is a comorbidity considered when evaluating for kidney transplantation. According to a study by Lenihan [15], over half of elderly candidates evaluated for a kidney transplant are not accepted due to their increased number of comorbidities.

Analyses of individuals denied for social reasons yielded our most alarming findings. African American and Hispanic patients were more likely to be denied for social reasons than White and Asian patients. Limited social support, insurance issues, limited transportation, and non-adherence are examples of such social reasons. Not only were these candidates more likely to be denied for social reasons, but non-adherence specifically appeared to be a factor that disproportionately affected African American candidates. Ninety-eight percent of all candidates who were denied due to non-adherence were African American. The term non-adherence is often used to describe a patient’s inability to adhere to a specific treatment plan as agreed upon with a physician, from the physician’s perspective. There are possible explanations for this finding. Due to the long-lasting impact of the Tuskegee Syphilis Study and others, many patients who identify as African American have ongoing mistrust of the medical community [16]. This can lead to hesitance to adhere to certain treatment plans set forth by members of the medical community. Therefore, we must work to build trust with our patient populations and continue current efforts to diversify our teams. Additionally, it has been shown that older African American patients experience more difficulty paying medical bills than older White patients [17]. Due to these difficulties, these patients may lack the ability to pay for specific devices, medications, or visits and are subsequently deemed non-adherent by their medical team.

This study was limited by its retrospective nature and by the volume of candidates who were evaluated in person compared to those evaluated via telemedicine during the peri-pandemic period. Due to constant changes and uncertainties during the pandemic, additional biases may have been introduced. Therefore, we intentionally chose a short observation time period to minimize differences in care. The relatively small number of patients in our cohort could also have contributed to some degree of type II statistical error. However, few other studies have examined the effects of the COVID-19 pandemic or telemedicine evaluations on access to listing for kidney transplant to date. In general, there is little focus on potentially modifiable factors that may limit access to the kidney transplant list for minority candidates prior to being listed. Additionally, there is currently no national database that specifically collects data on kidney transplant candidates prior to listing; thus, one strength of this study is the detailed characterization of reasons for listing or denial for listing available at a single institution, derived directly from electronic medical records.

## Conclusions

In summary, we found that while there were overall fewer evaluations in the peri-pandemic era, more candidates were listed for kidney transplant during that time. Telemedicine evaluations were associated with being listed for transplant. Race and ethnicity were not associated with being listed at our institution. However, better social support was associated with listing, but only in the pre-pandemic era. Curiously, employment and mental health status were similar in both eras despite pandemic stressors. Denial for listing due to social reasons affected mostly transplant candidates of minority ethnic groups, which warrants further study.
